# Effect of Aminosilane Coupling Agents with Different Chain Lengths on Thermo-Mechanical Properties of Cross-Linked Epoxy Resin

**DOI:** 10.3390/nano8110951

**Published:** 2018-11-19

**Authors:** Yujing Tang, Chao Tang, Dong Hu, Yingang Gui

**Affiliations:** College of Engineering and Technology, Southwest University, Chongqing 400715, China; 18382427662@139.com (Y.T.); losdot121@163.com (D.H.); yinganggui@swu.edu.cn (Y.G.)

**Keywords:** aminosilane coupling agent, cross-linked epoxy resin, molecular dynamics, thermo-mechanical properties

## Abstract

In this paper, a molecular dynamics simulation method was used to study the thermo-mechanical properties of cross-linked epoxy resins doped with nano silica particles that were grafted with 3-aminopropyltriethoxysilane, *N*-(2-aminoethyl)-3-aminopropyltrimethoxysilane, and 3-[2-(2-aminoethylamino)ethylamino]-propyl-trimethoxysilane with different chain lengths. Firstly, a set of pure epoxy resin models, and four sets of SiO_2_/EP composite models were established. Then, a reasonable structure was obtained through a series of optimizations using molecular dynamics calculations. Next, the mechanical properties, hydrogen bond statistics, glass transition temperature, free volume fraction, and chain spacing of the five models were studied comparatively. The results show that doped nano silica particles of surfaces grafted with 3-aminopropyltriethoxysilane, *N*-(2-aminoethyl)-3-aminopropyltrimethoxysilane, and 3-[2-(2-aminoethylamino)ethylamino]-propyl-trimethoxysilane with different chain lengths enhanced mechanical properties such as elastic modulus, shear modulus, and volume modulus obviously. The glass transition temperature increased by 15–16 K, 40–41 K, and 24–27 K, respectively. Finally, the data show that the cross-linked epoxy resin modified by nanoparticles grafted with *N*-(2-aminoethyl)-3-aminopropyl trimethoxysilane had better effects for improving thermo-mechanical properties by the comparatively studying the five groups of parameter models under the same conditions.

## 1. Introduction

Epoxy resin (EP) has good mechanical properties, thermal stability, and electrical properties [1,2]. It is widely used in power equipment such as dry-type transformers, insulating bushings, and basin-type insulator. In recent years, with the gradual increase in grid voltage class, the performance requirements of epoxy resin insulation material in all aspects are becoming increasingly demanding [3]. Therefore, it is necessary to improve the thermo-mechanical properties of epoxy resin. With the rapid development of nanotechnology, nanoparticles are widely used to improve the mechanical properties and toughness of polymers because of their surface and small size effect [4,5,6]. The characteristics of high hardness and corrosion resistance of nano silica particles [2] have resulted in their wide use by many domestic and foreign scholars as nano-modified fillers of polymer. However, nano silica particles are difficult to combine well with the resin matrix because of the high surface interaction energy and the large specific surface area. Nano silica particles are also easy to agglomerate when directly doped into the resin matrix. Therefore, this method failed to achieve the desired result [7,8]. In order to improve the dispersibility of nanoparticles in polymers, improve their compatibility with the resin matrix, and reduce the agglomeration of nanoparticles, a number of scholars have conducted experiments and research on these issues. Zhang et al. [9] grafted a silane coupling agent, with different grafting ratios, on nano silica particles, and then the mechanical properties of epoxy resin matrix with modified nanoparticles were studied. Chang et al. [10] experimentally grafted amino on nano silica and studied the effect of modified silica on the corrosion resistance of epoxy resin composites. Wang et al. [11] studied the effects of doped nano silica surface-grafted KH550 on the interfacial properties of epoxy resin. Li et al. [2] used a silane coupling agent as a bridging agent, which grafted bisphenol-A type epoxy resin and nanoparticles to improve the mechanical properties of the composites, while reducing the real and imaginary parts of the complex dielectric constant of the composite. There are many reports on the modification of inorganic nanoparticles by various surface treatment agents. Piscitelli et al. [12] experimentally studied the mechanical properties of montmorillonite (MMT) doped epoxy resin modified by different aminosilane coupling agents. The results of tensile stress, elongation at failure, and toughness showed that aminosilane coupling agents modified MMT-doped epoxy resin achieved good results. In Reference [13], the effects of aminosilane coupling agents, with different chain lengths modified MMT, on the properties of epoxy resin were studied by molecular simulation.

In this work, based on molecular dynamics, the effects of three aminosilane coupling agents, with different lengths, on the thermo-mechanical properties of cross-linked epoxy resin were studied. Firstly, a group of pure epoxy resin and four groups of SiO_2_/EP composite models were established. Then, the thermo-mechanical properties of cross-linked epoxy resins were predicted by microscopic indexes such as elastic modulus, volume modulus, shear modulus, hydrogen bonds, glass transition temperature, free volume fraction, and chain spacing. Finally, the aminosilane coupling agent with the best improving properties was selected.

## 2. Materials and Methods

**Figure 1:**
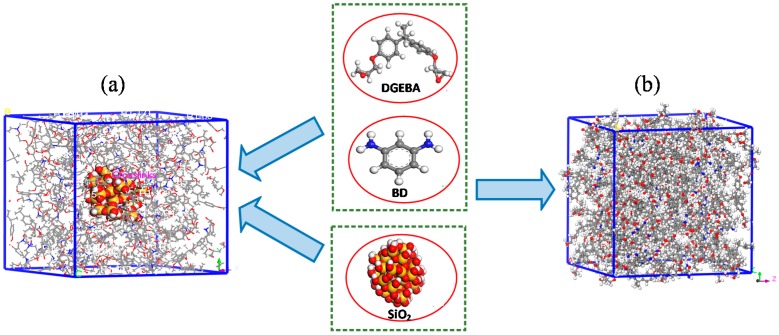
(**a**) SiO_2_ composite model; (**b**) pure epoxy resin model.

**Figure 2:**
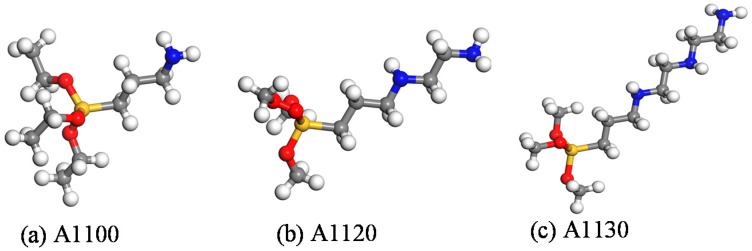
Three different chain length aminosilane coupling agents; (**a**) 3-aminopropyltriethoxysilane; (**b**) *N*-(2-aminoethyl)-3-aminopropyltrimethoxysilane; (**c**) 3-[2-(2-amino-ethyl) Ethylamino)ethylamino]propyl-trimethoxysilane.

**Figure 3:**
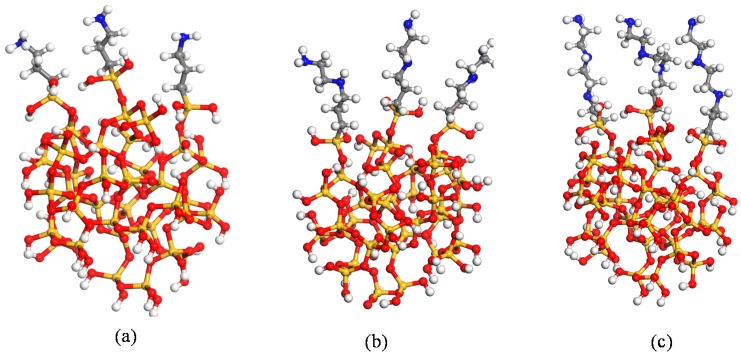
(**a**) A1100 aminosilane coupling agents grafted on the surface of nanoparticles; (**b**) A1120 aminosilane coupling agents grafted on the surface of nanoparticles; (**c**) A1130 aminosilane coupling agents grafted on the surface of nanoparticles.

In this paper, molecular dynamics simulations were performed using Materials Studio software. The pure epoxy resin model, the EP/SiO_2_ composite model (the surface of SiO_2_ without any modifier), and surface of the silica respectively grafted with epoxy resin doped with 3-aminopropyltriethoxysilane (A1100), *N*-(2-aminoethyl)-3-aminopropyltrimethoxysilane (A1120), and 3-[2-(2-aminoethylamino)ethylamino (A1130) were built, which were represented as pure, SiO_2_, A1100, A1120, and A1130. In this paper, COMPASS [11,14,15,16,17,18] was selected as the force field for calculations in all dynamic simulations; Nose [18] and Berendsen [19] were used for temperature control and pressure control, respectively. Figure 1 shows the SiO_2_ composites and the pure epoxy resin model. The specific steps to establish the models were as follows:

(1) Bisphenol A epoxy resin (DGEBA) and 1,3 benzenediamine (BD) hardener monomer were constructed by Forcite module, and a box with the 50 × 50 × 50 Å^3^ was built using Build Tool, then DGEBA and BD molecules were added in a ratio of 2:1 into it. The initial density was 0.6 g/cm^3^ [9], and then a three-dimensional periodic cell model was built. Next, the model was geometrically optimized using the 10,000-step iterations to obtain the lowest energy conformation. Subsequently, molecular kinetic calculations for constant volume and temperature (NVT)ensemble and constant pressure and temperature (NPT) ensemble were performed. Afterwards, the last equilibrated unit cell was cross-link modeled by scripts (perl). The cross-linking reaction was performed between the R1 carbon atoms on the pre-labeled epoxy resin of DGEBA molecules, and the R2 nitrogen atoms labeled on the curing agent BD molecules. It is presupposed that the degree of crosslinking is 100%, but in general, the final result is less than 100% [15,20].

(2) The mechanical properties of cross-linked epoxy resins were affected by the size of nanoparticles [21,22], so spherical silica nanoparticles with a radius of 0.66 nm were constructed in this paper, and the surfaces of nano silica particles were hydrogenated [23].

(3) The aminosilane coupling agents with different chain lengths were grafted on the nanoparticles before being constructed in step (2). Figure 2 shows the aminosilane coupling agent with different chain lengths without any dealt. First, three kinds of silane coupling agents underwent a hydrolysis reaction in the actual reaction process, and then the three aminosilane coupling agents A1100, A1120, and A1130 were grafted onto the nanoparticles after hydrogenation, which are shown in Figure 3. The grafting densities of the three silane coupling agents were the same. Before the nanoparticles were built, they were placed in the center of a crystal cell box of 50 × 50 × 50 Å^3^, then the cross-linked modeling could be obtained by repeating step (1). Finally, the NVT and NPT were conducted at 300 K to obtain a global equilibrium structure.

## 3. Results

### 3.1. Mechanical Properties

In general, the stress-strain behavior in linear-elastic materials [20] of solid materials can be obtained by Hooke’s law, which is expressed as follows:

(1) $$\sigma_{i}=C_{ij}\varepsilon_{j}$$

$C_{ij}$ is the six-dimensional stiffness matrix, $\sigma_{i}$ and $\varepsilon_{j}$ are the stress and strain vectors respectively, the stress component can be expressed as follows:

(2) $$\sigma_{\mathrm{ij}}=-\frac{1}{V}\sum_{k}[m^{k}(u_{i}^{k}u_{j}^{k})]+\frac{1}{2}\sum_{i\neq k}(r_{i}^{kl})f_{j}^{lk}$$

where *V* stands for volume, *m^k^* and *u^k^* are the mass and velocity of the *k*th particle, $r^{kl}$ indicates the distance between the *k*th and the lst particle, $f^{lk}$ indicates the force applied between the *k*th and the 1st particle. The lame coefficients λ and μ could be calculated as follows [24]:

(3) $$\lambda =\frac{1}{6}(C_{12}{+C}_{13}+C_{21}+C_{23}+C_{31}+C_{32})\approx \frac{1}{3}(C_{12}{+C}_{23}{+C}_{13})$$

(4) $$\mu =\frac{1}{3}(C_{44}+C_{55}+C_{66})$$

(5) $$\lambda +2\mu =\frac{1}{3}(C_{11}+C_{22}+C_{33})$$

The elastic modulus (*E*), volume modulus (*K*), shear modulus (*G*) can be obtained using the following equations:

(6) $$E=\frac{\mu (3\lambda +2\mu )}{\lambda +\mu }$$

(7) $$K=\lambda +\frac{2}{3}\mu$$

(8) G=μ

**Figure 4:**
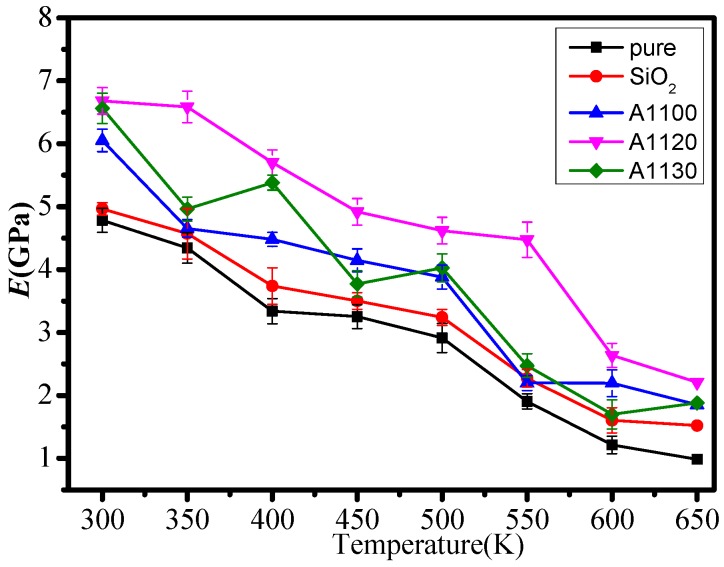
Elastic modulus of five models at different temperatures.

**Figure 5:**
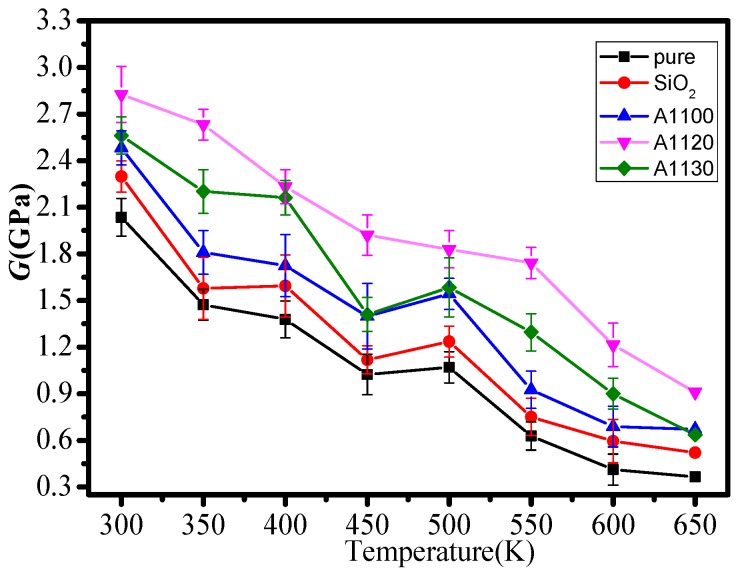
Shear modulus of five models at different temperatures.

**Figure 6:**
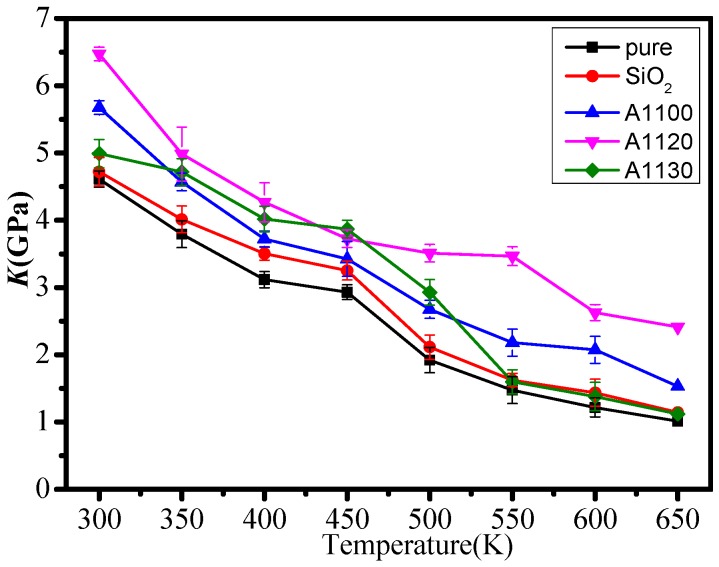
Volume modulus of five models at different temperatures.

In this paper, the mechanical performance parameters of five models at 300–650 K were calculated. Figure 4, Figure 5 and Figure 6 show the elastic modulus (*E*), shear modulus (*G*), and volume modulus (*K*) of the five statistical models at different temperatures. The mechanical properties of several models fluctuated with the rise in temperature, which was consistent with previous reports [20]. Meanwhile, the elastic modulus, shear modulus, and volume modulus of the five models all decreased gradually with the increase in temperature. By comparing the mechanical properties of the five group models, it can be found that the mechanical properties of epoxy resin doped with nanoparticles at the same temperature are better than those of unmodified epoxy resin. It indicates that the mechanical properties of epoxy resin modified by nanoparticles are effective. At the same time, it is known that the mechanical properties of the A1100, A1120, and A1130 composite models are better than those of the EP and SiO_2_ models. The results show that it is necessary to graft silane coupling agents on the surface of nanoparticles to improve the mechanical properties of epoxy resin. In the three groups of nanoparticles grafted with aminosilane coupling agents, the mechanical properties of the A1120 model were significantly higher than those of the A1100 and A1130. Moreover, with the increase in temperature, the mechanical parameters of the A1120 model declined slightly, indicating that the mechanical properties of the A1120 modified nano silica doped epoxy resin were better than the A1100 and A1130.

### 3.2. Hydrogen Bond Calculation

**Figure 7:**
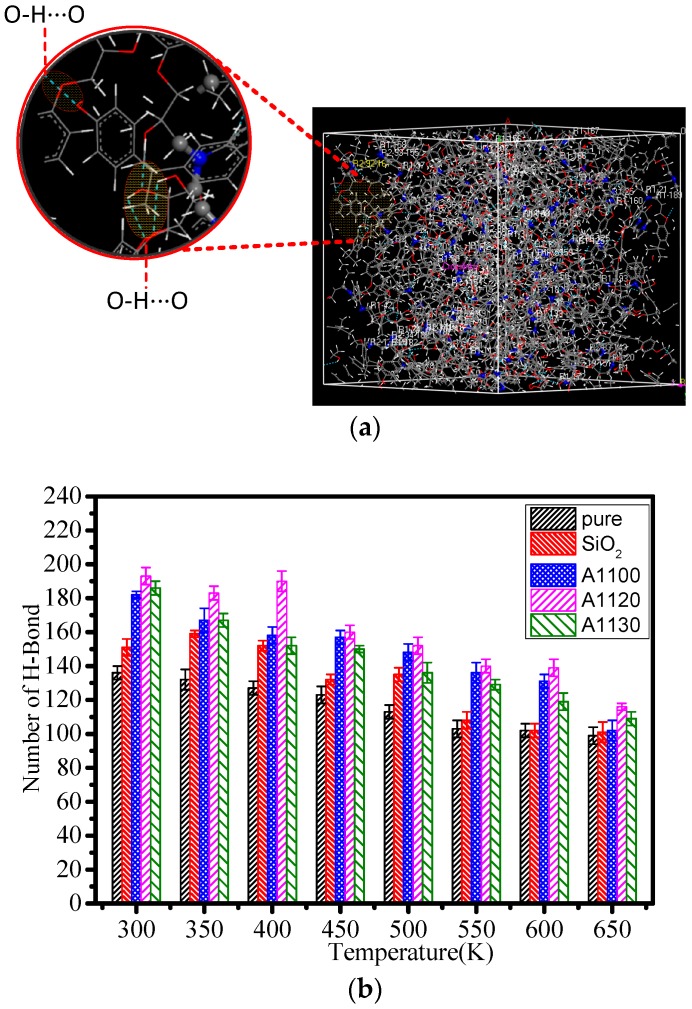
(**a**) Schematic diagram of the hydrogen bond formed by the cross-linked epoxy resin, in the form of a blue dotted line in the yellow shade, the hydrogen bond, here is the OH⋅⋅⋅Htype hydrogen bond; (**b**) number of hydrogen bonds at different temperatures.

Hydrogen bonding generally refers to a bond formed between a highly electronegative atom in a molecule and another electronegative atom, which produces a certain binding force through the H atom. In classical mechanics definition of hydrogen bond [25,26], it has two modes: Energy criterion and geometric criterion. The literature [27] shows that when the interaction between molecular pairs is stronger than a certain value, this is an indication that hydrogen bonds will exist between the molecules. In the geometric principle, the existence of intermolecular hydrogen bonds depends on the relative position of two molecules [28,29]. The hydrogen bond can be expressed as “X-H⋅⋅⋅Y”, X and Y represent the two electronegativity elements, where X and Y are donor and acceptor. In general, a hydrogen atom can form one to two hydrogen bonds, and the hydrogen bond is defined mainly by geometric principles in this paper, Figure 7a shows a schematic diagram of a hydrogen bond.

Figure 7b shows that the number of hydrogen bonds in the five models decreased with increasing temperatures. This is due to the fact that the structure of epoxy resin will change with the increase in temperature, and the speed of molecule movement will accelerate, leading to an increase in the distance between molecules. Therefore, the formation condition of partial hydrogen bonds was broken, and the result was that the number of hydrogen bonds was drastically reduced. So, the reduction of hydrogen bonds will inevitably lead to a decrease in the toughness and chemical stability of epoxy resin [30]. Under the same conditions, the number of hydrogen bonds of the A1100, A1120, and A1130 was significantly higher than that of pure and SiO_2_. At the same time, the number of pure hydrogen bonds is obviously the smallest. Among the three models of grafting the aminosilane coupling agents to the surface of nanoparticles, the A1120 had the most hydrogen bonds at the same temperature, A1100 was the second, and A1130 was the smallest. The results show that the toughness of the epoxy resin was enhanced by doping nanoparticles. Furthermore, the mechanical properties of the cross-linked epoxy resin were improved by grafting A1130 onto the surface of doped nanoparticles, but the toughness was sacrificed compared with the grafted A1100.

### 3.3. Glass Transition Temperature Analysis

**Figure 8:**
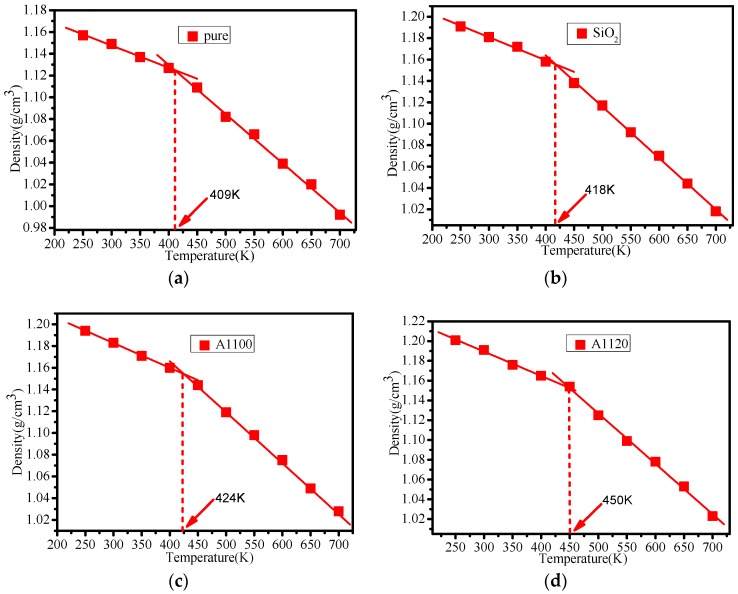

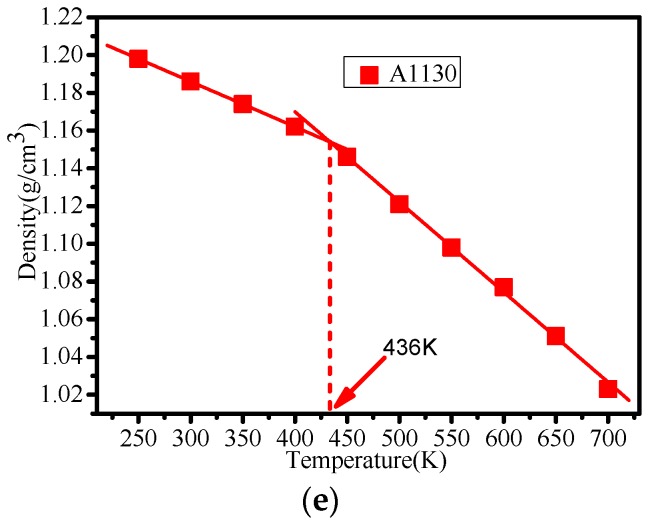
(**a**) The density-temperature fitting curve of the pure model; (**b**) the density-temperature fitting curve of the SiO_2_ model; (**c**) the density-temperature fitting curve of the A1100 model; (**d**) the density-temperature fitting curve of the A1120 model; (**e**) the density-temperature fitting curve of the A1130 model.

The glass transition temperature (*T_g_*) is a significant parameter that reflects the thermal properties of polymer materials. When the temperature is below *T_g_*, the material is in a glassy state, and when the temperature is above *T_g_,* the material is in a high elastic state. In general, the transition state between the glass state and the high elastic state is called the glass transition, and the corresponding temperature at the time of the transition is called the glass transition temperature. The glass transition temperature can be obtained by linear fitting of density and temperature [20,31]. Likewise, *T_g_* can also be obtained by fitting curves of free volume and temperature [32]. Figure 8 shows the glass transition temperature obtained by fitting the density-temperature curve. The calculated glass transition temperatures of pure, SiO_2_, A1100, A1120, and A1130 were 409 K, 418 K, 424 K, 450 K, and 436 K, respectively. From the statistical results, the glass transition temperatures of the grafted silane coupling agent models were significantly better than those of the pure epoxy resin and SiO_2_ models. Among them, the A1120 had the highest glass transition temperature, where the *T_g_* of A1100, A1120, and A1130 increased by 15 K, 41 K, and 27 K respectively compared with pure epoxy resin. The results indicate that the aminosilane coupling agents could improve the thermal properties of the cross-linked epoxy resin. In some examples within the literature [9,11,32,33,34], the glass transition temperature shifted *T_g_* from 400 K to 483 K. Different epoxy resin monomers, curing agents, and experimental conditions make the *T_g_* different to some extent, which indicates that the simulation in this paper has certain reliability.

### 3.4. Free Volume Calculation

The free volume not only can reflect the stacking capacity of cross-linked epoxy resin molecules, but also predicts thermal and mechanical properties [9]. In this paper, the Atom Volume & Surface tool of Materials Studio software was used to measure the free volume of several models at different temperatures. According to the theory of Fox and Flory [35], the volume of the system can be divided into free volume (*V_f_*) and occupied volume (*V_o_*). The free volume fraction can be calculated as follows:

(9) $$FFV=\frac{V_{f}}{V_{f}+V_{o}}\times 100\%$$

Table 1 reflects the calculated free volume fraction of the five models at different temperatures. From the statistical results, the free volume fractions of A1100, A1120, and A1130 were lower than those of pure and SiO_2_, and the free volume fraction of cross-linked epoxy without any nanoparticles was the most. The value of the free volume fraction increased with the increase in temperature, which reflects an increase in the molecular chain motion space. However, the large free volume fraction would result in a decrease in the thermo-mechanical properties of epoxy resin [9]. At the same time, a consistent finding was obtained that the A1120 have the smallest free volume fraction under the same conditions. This result shows that the properties of cross-linked epoxy resin modified by nanoparticle surfaces grafted with the A1120 silane coupling agent were better than those of A1100 and A1130, which is consistent with previous studies. In addition, it can be found that the free volume fraction of the A1130 in the glass state was lower than that of the A1100. When in the rubber state, the free volume fraction of A1130 was higher than that of the A1100. Overall, it is necessary to dope nanoparticles to improve the thermal and mechanical properties of epoxy resin, and nanoparticles modified by A1120 had better effects.

**Figure 9:**
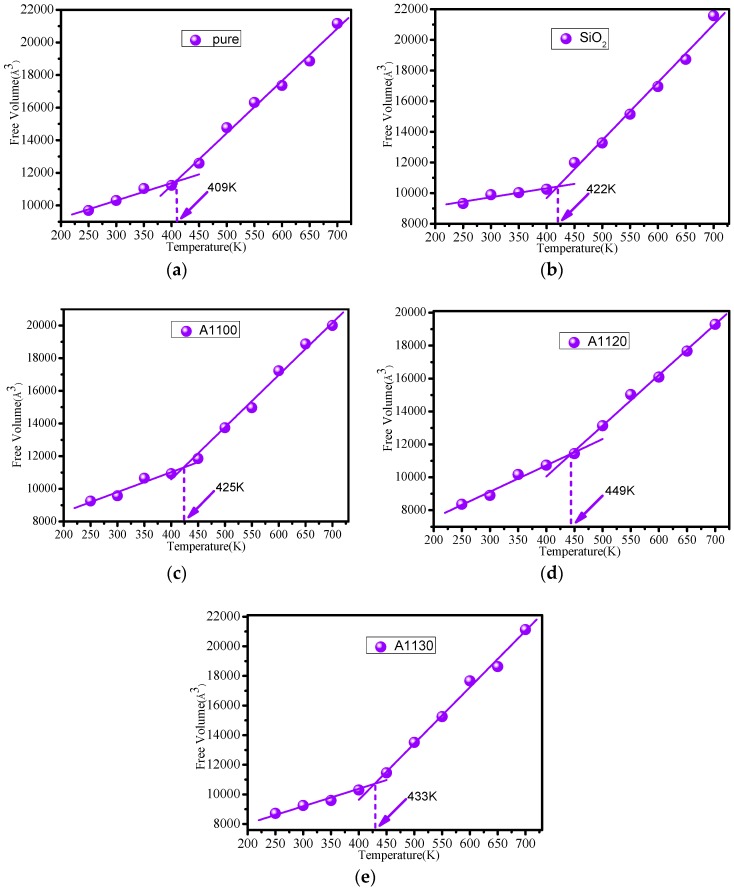
(**a**) Free volume-temperature fitting curve of pure epoxy resin model; (**b**) free volume-temperature fitting curve of SiO_2_ model; (**c**) free volume-temperature fitting curve of A1100 model; (**d**) free volume-temperature fitting curve of A1120 model; (**e**) free volume-temperature fitting curve of A1130 model.

Figure 9 shows the glass transition temperature curve of the cross-linked epoxy resin obtained by fitting the free volume and temperature. When the temperature was below the glass transition temperature, which is in the glass state, the value of free volume of epoxy resin was small. While when the temperature exceeded the glass transition temperature, which is in the rubber state, the free volume of epoxy resin increased rapidly with the rise in temperature. The rise of free volume promotes the acceleration of molecular chain movement. The glass transition temperatures of pure, SiO_2_, A1100, A1120, and A1130 obtained by fitting the free volume-temperature curves were 409 K, 420 K, 425 K, 449 K, and 433 K, respectively. *T_g_* of A1100, A1120, and A1130 obtained by volume-temperature fitting curve enhanced by 16 K, 40 K, and 24 K, respectively. This result was close to the glass transition temperature obtained from the previously calculated density-temperature fitting curve. Therefore, the reliability of the calculations in this paper is demonstrated again.

### 3.5. Polymers Chain Spacing Calculation

In this paper, in order to study the reason why the doped nanoparticles grafted with aminosilane coupling agents of different lengths caused different thermos-mechanical properties of the cross-linked epoxy resin, X-ray was used to study the chain spacing of A1100, A1120, and A1130 models at the temperature of 300 K. The chain spacing of the composite reflects the stacking state of the polymer chain [36]. When X-ray diffraction (XRD) passes through polymer, the chain spacing of the polymer can be obtained by the following formula:

(10) $$d=\frac{\lambda }{2\sin\theta }$$

**Figure 10:**
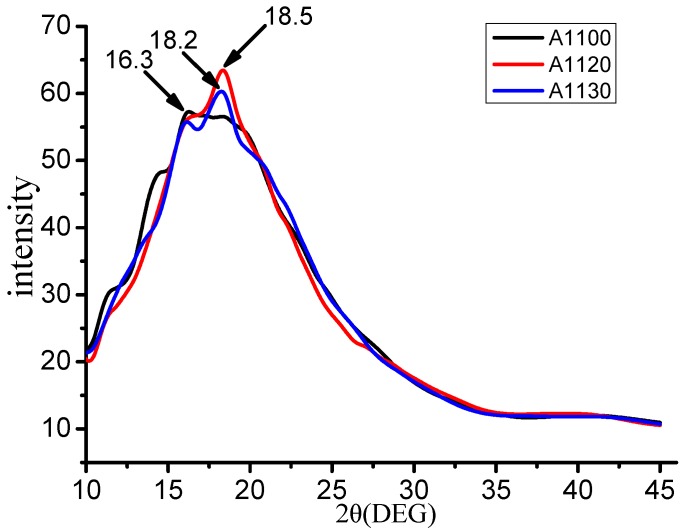
X-ray diagram of compound model of silane coupling agents with different chain length.

Using the scattering function in Forcite and X-ray as radioactive source, XRD spectra is calculated. The diffraction parameters were set as follows: The range of 2*θ* and cutoff was 5~45 Å and 50 Å, respectively. Where λ is the wavelength of the X-ray, here it is 0.154 nm, and *θ* is the diffraction angle corresponding to the peak of the diffraction curve. Figure 10 shows the X-ray diffraction pattern of cross-linked epoxy resin doped with nano silica particles grafted on the surfaces of three silane coupling agents of A1100, A1120, and A1130, respectively. According to Figure 10, the 2*θ* of A1100, A1120, and A1130 were 16.3 degrees, 18.5 degrees and 18.2 degrees.

The increase of chain spacing of polymers would make the density decrease and the hydrogen bond break. The results of the chain spacing calculated from Table 2 show that the order of the chain spacing of the three models is A1100 > A1130 > A1120. It is further proved that the cross-linked epoxy doped with nano silica particles surface grafted A1120 has better effects of improving thermo-mechanical properties. Why aminosilane coupling agents of A1120 modified nano silica particles doped cross-linked epoxy resin have better thermo-mechanical properties than those of A1100 and A1130? The possible reasons could be explained as follows: amino groups of the aminosilane coupling agents can modify nanoparticles, enhance the dispersion of nano silica particles, reduce the agglomeration of nanoparticles, and make nanoparticles effectively react with the epoxy resin matrix. Ultimately the thermo-mechanical properties of epoxy resin were improved. The amino chain length of A1120 is longer than that of A1100, which makes A1120 have a better effect on improving thermo-mechanical properties. With the further increase of the amino chain length of A1130, the excessive amino chain length of A1130 will limit the movement of nano silica particles, so that nanoparticles could not completely react with the epoxy resin matrix, leading to the degradation of the thermo-mechanical properties of the epoxy resin.

## 4. Conclusions

In this paper, the thermo-mechanical properties of cross-linked epoxy resin doped with nano silica particles were studied using molecular dynamics methods. The effects of nano silica particles surface grafted with three aminosilane coupling agents with different chain lengths, which were doped into cross-linked epoxy resin were comparatively studied. The obtained conclusions are as follows:

Doping nano silica particles can improve the mechanical properties and thermal stability of cross-linked epoxy resin. Moreover, the simulation results showed that nanoparticles surface grafted with aminosilane coupling agents into cross-linked epoxy resin can effectively improve the mechanical properties and thermal stability of cross-linked epoxy resin. Nanoparticles grafted with three amino-silane coupling agents with different chain lengths did not only effectively improve the mechanical properties such as elastic modulus, shear modulus, and volume modulus, but also improved thermal stability such as enhancing the toughness effectively, and increasing the *T_g_* when compared to the pure epoxy resin model and epoxy resin model directly doped by untreated SiO_2_. Finally, an obvious result was obtained showing that among the three aminosilane coupling agents with different chain lengths, the cross-linked epoxy resin modified by nanoparticles grafted with *N*-(2-aminoethyl)-3-aminopropyl trimethoxysilane had better effects for improving thermo-mechanical properties, which may provide a reference for subsequent modifications of aminosilane coupling agents.

## Figures and Tables

**Table 1:** Calculation of free volume fraction (100%).

Temperature (K)	250	300	350	400	450	500	550	600	650
pure	0.151	0.159	0.168	0.170	0.187	0.212	0.231	0.246	0.290
SiO_2_	0.148	0.156	0.157	0.176	0.183	0.198	0.203	0.233	0.260
A1100	0.148	0.151	0.166	0.173	0.178	0.196	0.217	0.246	0.263
A1120	0.135	0.142	0.160	0.167	0.182	0.197	0.220	0.240	0.251
A1130	0.141	0.148	0.149	0.168	0.184	0.200	0.220	0.249	0.259

**Table 2:** Chain spacing of the cross-linked epoxy resins doped nano silica particles grafted with three different chain lengths of aminosilane coupling agents.

System	2*θ* (°)	d-Spacing (Å)
A1100	16.3	5.43
A1120	18.5	4.79
A1130	18.2	4.87
